# Forehead Erythematous Rash: A Rare Manifestation of Tick-Borne Lymphadenopathy (TIBOLA)

**DOI:** 10.7759/cureus.86056

**Published:** 2025-06-15

**Authors:** Francisca Bartilotti Matos, Rita de Sousa, Luís Malheiro

**Affiliations:** 1 Infectious Diseases Department, Centro Hospitalar, Vila Nova de Gaia, PRT; 2 Center for Vector and Infectious Diseases, Instituto Nacional de Saúde Dr. Ricardo Jorge, Lisbon, PRT; 3 Infectious Diseases Department, Centro Hospitalar, Vila Nova De Gaia, PRT

**Keywords:** infectious and tropical diseases, tibola, tick-borne diseases, tick-borne infections, tropical medicine

## Abstract

Tick-borne lymphadenopathy (TIBOLA) is a rickettsial infection caused by *Rickettsia slovaca*. It has been increasingly identified across Europe. We present the case of TIBOLA with a unique erythematous forehead rash, which hasn't been described before. Four days after a tick bite on the scalp, a woman presented to the emergency department with a painful swelling in the right retroauricular area and a headache. Examination revealed a palpable lymph node and fever. No eschar was observed. Initial serology for common tick-borne infections was negative. Three days later, she returned to the hospital with an erythematous rash on her forehead. Despite not receiving antibiotic treatment, the symptoms resolved spontaneously. A follow-up serum sample, taken six weeks later, showed seroconversion for spotted fever group rickettsia, leading to the diagnosis of TIBOLA. TIBOLA often goes unrecognized due to nonspecific symptoms and the hidden location of eschars. This case emphasizes the importance of considering TIBOLA in tick-borne illness diagnoses in Portugal.

## Introduction

Tick-borne lymphadenopathy (TIBOLA), also known as *Dermacentor*-borne necrosis erythema and lymphadenopathy (DEBONEL) and scalp eschar and neck lymphadenopathy after a tick bite (SENLAT), is a rickettsial infection transmitted by ticks [1]. The causative agent, *Rickettsia (R.) slovaca*, was first identified in France in 2002 [2]. It is a member of the spotted fever group (SFG) *Rickettsia, *such as *R. conorii*, *R. africae,* or *R. rickettsii*. Over the years, it has been reported across several European countries [3,4]. It is considered to be the second most prevalent rickettsiosis in Europe, after Mediterranean Spotted Fever [3], with over 200 cases reported in Spain [5]. This syndrome can, though less commonly, be caused by other microorganisms, such as *Candidatus R. rioja*, *R. massiliae*, *R. sibirica mongolitimonae*, *Bartonella henselae*, and *Francisella tularensis* [2]. In Portugal, *R. slovaca* was first identified in *Dermacentor (D.) *ticks in 1995 [1]. According to the Portuguese Nationwide Surveillance Program on Vector and Vector-Borne Diseases (REVIVE), *D. marginatus* and *D. reticulatus* ticks have a widespread presence in the country, with peak activity in spring and autumn. These ticks were found to carry *R. slovaca*, *Candidatus R. rioja*, and *R. raoultii* [6]. 

Like other rickettsial diseases, the causative agents are inoculated by the tick, targeting macrophages and dendritic cells and then spreading through the lymphatic system to the regional lymph nodes [7]. TIBOLA's incubation period typically lasts 5 to 10 days [8]. Common symptoms include an inoculation eschar, fever, rash, and painful, enlarged lymph nodes. Other symptoms can include fatigue, scarring alopecia, and vertigo [9,10]. Generally, the disease is mild, although severe cases, such as myopericarditis, have been reported [11]. Diagnosis relies on clinical signs and epidemiological factors [12]. The recommended treatment is a 7-10 day course of doxycycline [4].

We present a case report of TIBOLA diagnosed in northern Portugal with a novel clinical presentation.

## Case presentation

We report a case of a woman in her fifties with a medical history of asthma and hypertension. She presented to the emergency department (ED) in April with right-sided neck pain that worsened with movement, accompanied by a painful swelling in her right retroauricular area and a burning headache. She recalled a tick bite to her scalp four days earlier. A tick bite had also been identified on her daughter's scalp two days earlier. Physical examination revealed no inoculation eschar, but a swollen, 1-2 cm lymph node in her right retroauricular area and a fever of 38.2ºC. Initial tests showed a normal blood count, renal function, and ionogram, with no rise in C-reactive protein. Serological tests for *Rickettsia conorii*, *Borrelia spp.*, and *Coxiella burnetii* and molecular detection of Rickettsial DNA were also negative.

**Figure 1:**
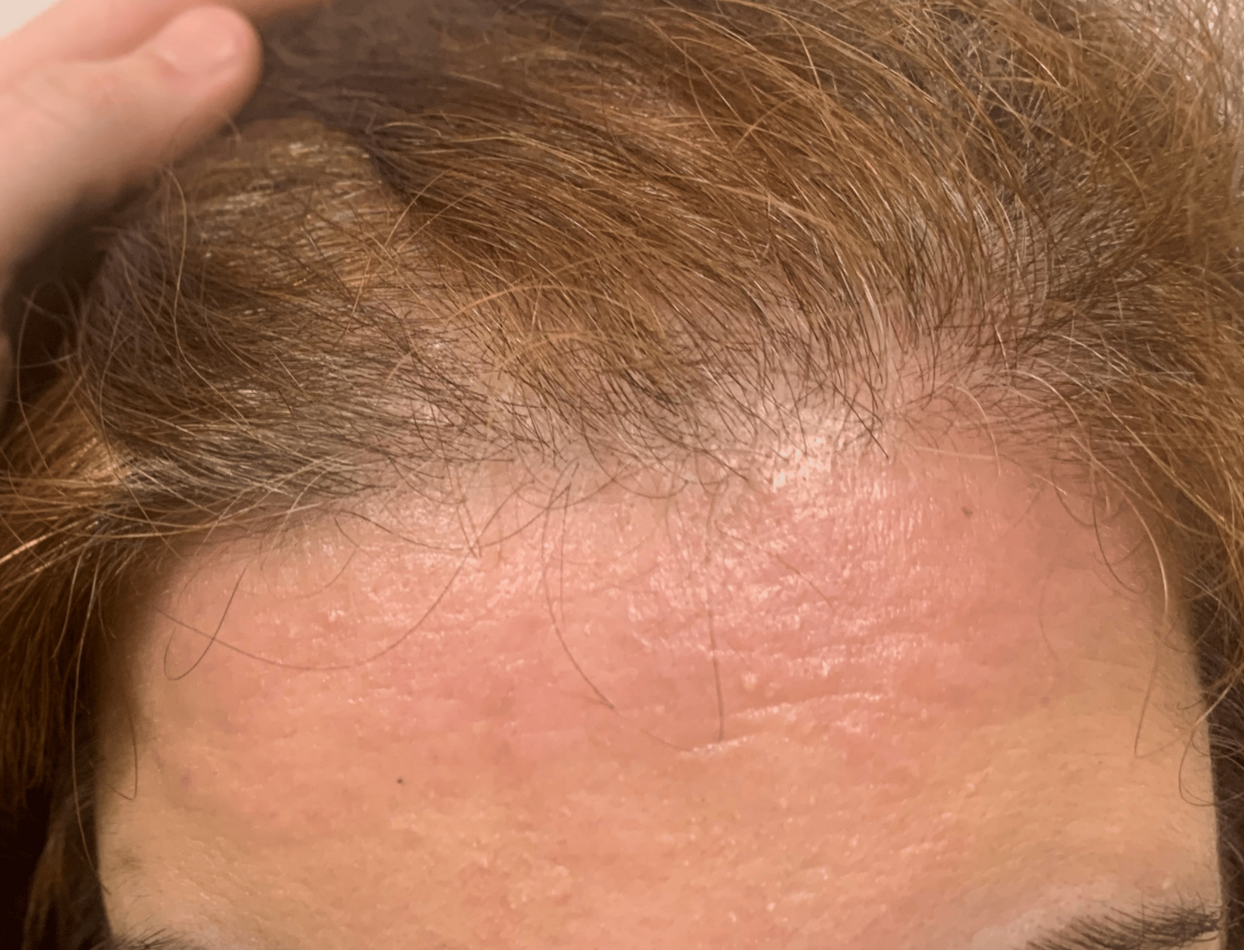
Pruriginous erithematous rash in the forehead after tick bite - a novel presentation of TIBOLA TIBOLA - tick-borne lymphadenopathy

She was discharged with symptomatic treatment (paracetamol). She returned to the ED three days later, due to the appearance of a pruriginous erythematous rash on her forehead (Figure 1).

**Figure 2:**
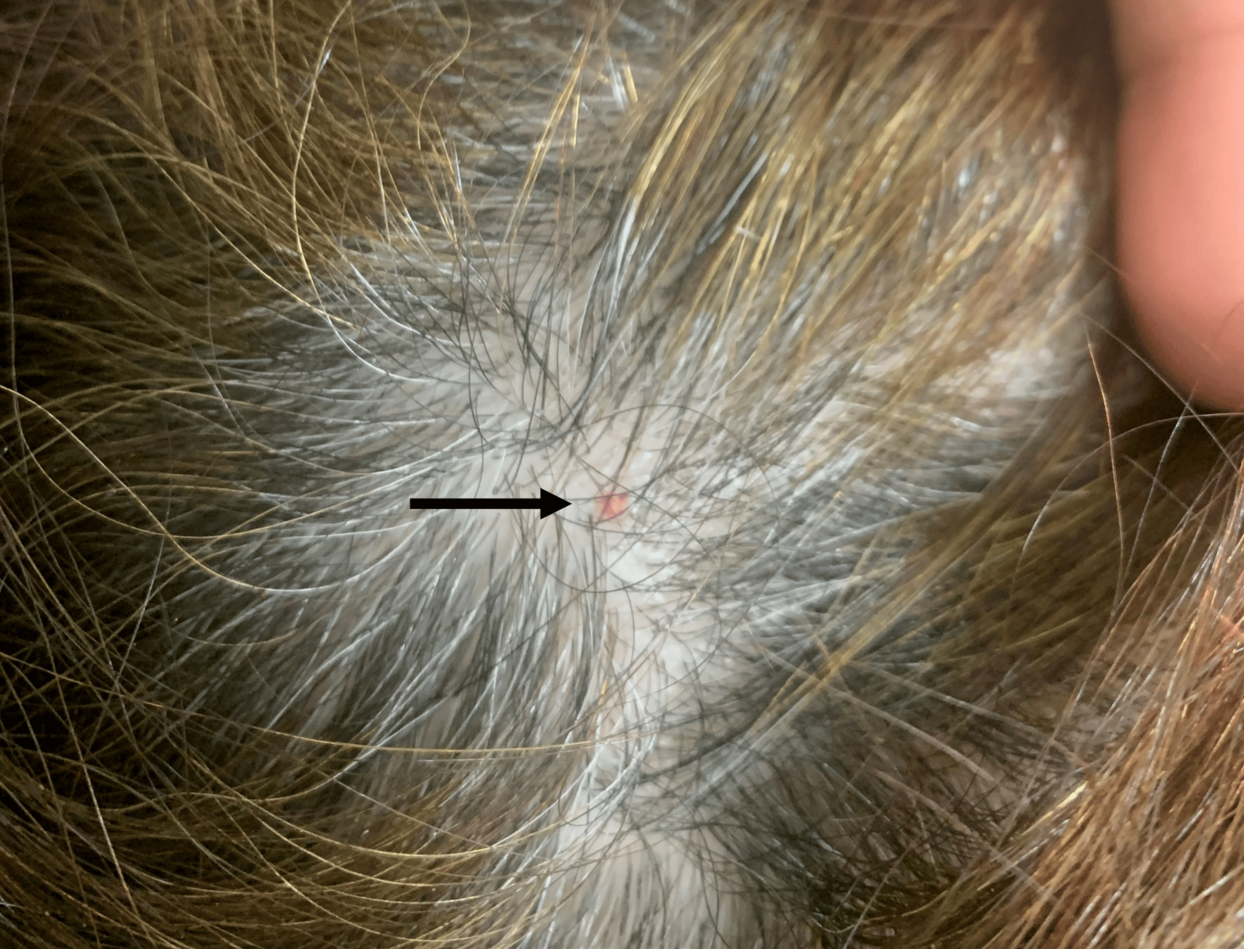
A small red macula (arrow) was observed at the site of tick removal, in the occipital area of the scalp

A small red macula was observed at the site where the tick was removed (Figure 2).

Her fever had subsided, but the lymph node remained swollen. She was referred for follow-up at the infectious diseases clinic, where further testing was conducted, and she was medicated with antihistamines. At the six-week follow-up appointment, serological testing using an indirect immunofluorescence assay (IFA) for Rickettsia showed seroconversion, with reactive titers of ≥ 256 for IgM (cut-off titer ≥ 32) and 2048 for IgG (cut-off titer ≥ 128) against SFG *Rickettsia *(*R. conorii*) (Table 1).

**Table 1:** Comparison of serology results between the emergency department visit and the six week follow up Results show a fourfold increase in IgM/IgG serology for SFG Rickettsia. IFA was performed by using the commercial BIOCELL kit slides. SFG - spotted fever group; IFA: immunofluorescence assay

	Emergency department visit	6-week follow up	Reference value
*Rickettsia conorii* (SFG *Rickettsia*) IgG (IFA)	128	2048	≥ 128
*Rickettsia conorii* (SFG *Rickettsia*) IgM (IFA)	<32	≥ 256	≥ 32
*Coxiella burnetii* IgG (IFA)	<1/16	<1/16	≥1/16
*Coxiella burnetii* IgM (IFA)	<1/16	<1/16	≥1/16
*Borrelia spp *IgG	0.01	0.01	Negative: < 2.0 Positive: ≥ 2.0
*Borrelia spp *IgM	0.00	0.00	Negative: < 0.20 Dubious: 0.20 - 0.31 Positive: ≥ 0.32

The IFA was performed using commercial BIOCELL kit slides. By the time results were available, the patient's symptoms had resolved. She was contacted three months later and reported no symptom recurrence and no long-term sequelae. Her daughter, who was also bitten by a tick, did not develop any symptoms. 

Due to serological cross-reactivity within the SFG Rickettsia, Rickettsia species causing the illness could not be identified [5].

However, considering the patient's history and symptoms, combined with their improvement without specific antibiotic treatment and a fourfold increase in IgM/IgG serology for SFG Rickettsia, TIBOLA was considered the most likely diagnosis. After discussion with the team and the patient, and due to the mild, self-limited nature of her symptoms, it was considered that treatment with doxycycline would be unnecessary.

## Discussion

TIBOLA has a seasonal incidence pattern, peaking in spring and autumn, and affects more women and children [13]. Due to its non-specific symptoms and the often concealed location of inoculation eschars, the diagnosis of TIBOLA is frequently delayed [8]. This case reflects the disease's seasonality and typical patient demographic. Unlike TIBOLA, Mediterranean spotted fever peaks in summer due to the seasonal peak activity of *Rhipicephalus sanguineus*, the main vector [9]. A literature review of TIBOLA cases in Portugal identified one case series of three patients and one isolated case report (Table 1). They all referred to patients in the central region of the country.

**Table 2:** Characterization of previous case reports of TIBOLA/DEBONEL/SENLAT in Portugal NA - not available; ND - not done; PCR - polymerase chain reaction; TIBOLA - tick-borne lymphadenopathy; DEBONEL  - Dermacentor-borne necrosis erythema and lymphadenopathy; SENLAT - scalp eschar and neck lymphadenopathy after a tick bite

	Patient one (Sousa et al., 2013 [1])	Patient two (Sousa et al., 2013 [1])	Patient three (Sousa et al., 2013 [1])	Patient dour (Quadros Flores et al., 2022 [14])
Year	2010	2012	2012	2024
Age	50	53	30	37
Sex	Female	Female	Female	Female
Season	Autumn	Spring	Spring	Spring
Region	Coimbra (centre)	Coimbra (centre)	Coimbra (centre)	Guarda (centre)
Incubation period	4 days	7 days	NA	10 days
Eschar present	Yes	Yes	Yes	Yes
Fever	Yes	No	No	No
PCR from eschar	Yes	Yes	Yes	No
Serology seroconversion	Yes	Yes	ND	Yes

Despite the absence of an eschar, this patient's tick bite, fever, and unilateral painful lymphadenopathy were consistent with TIBOLA symptoms.

Interestingly, cutaneous manifestations of TIBOLA have included facial oedema and macular lesions of the extremities [12]. We have not found a previous description of a pruriginous erythematous rash localised to the forehead in previous case reports. This constitutes a novel presentation that could be a helpful clue to diagnosing further cases.

Although serology testing has limitations, including significant cross-reactivity among Rickettsia species within the SFG and low sensitivity in the acute phase of the disease. Seroconversion, demonstrated by a four-fold increase in antibodies, remains the gold standard for diagnosing recent rickettsial infections [10,15]. Given the patient's symptoms, the infection was likely caused by *R. slovaca* or *R. raoultii*. The initial negative IgM result is consistent with expectations, as IgM antibodies may take up to 14 days to become detectable [15,16]. The fact that the infection resolved without any antibiotic treatment is also more consistent with milder rickettsial infections, such as TIBOLA. In retrospect, doxycycline could have been initiated at the time of the presentation to the ED, as the patient's history and symptoms were consistent with a rickettsiosis, and timely initiation of treatment is recommended [10]. Though antibiotic treatment is not always necessary, as seen in this case, it does contribute to shortening the duration of symptoms and lowering the risks of long-term sequelae [9].

Unfortunately, molecular detection by polymerase chain reaction (PCR) testing was negative, which hinders the identification of the specific Rickettsia species. PCR for rickettsial infections has limited sensitivity due to the low rickettsiaemia, particularly in localized syndromes such as TIBOLA [16,17], with a case series in France reporting a sensitivity of only 50% [18]. It is not uncommon for TIBOLA cases to lack a clearly identified aetiological agent. A review found that the aetiology was determined in only 149 of 537 cases described in the literature [9]. 

Reported as an emerging infection, TIBOLA has been identified in various regions of Portugal, although all previous case reports have been from central Portugal [1]. The distribution of both the tick vector and pathogen suggests that TIBOLA may be more prevalent than is currently recognized, highlighting the importance of raising clinical awareness of this rare disease [1,15].

## Conclusions

This case reports an unusual presentation of TIBOLA, with an erythematous rash on the forehead. TIBOLA should be considered in patients presenting with fever, painful lymphadenopathy, and localized rashes after a tick bite, even if an eschar is absent. Seroconversion with a fourfold increase in IgM/IgG antibodies is the gold standard for diagnosing rickettsial infections, highlighting the importance of follow-up serological testing. Low rickettsial levels in infections like TIBOLA may lead to negative PCR results from blood samples. PCR testing of eschar or lymph node biopsies can improve diagnostic accuracy for identifying the Rickettsia species involved.
